# Amino acid transporter SLC7A11/xCT at the crossroads of regulating redox homeostasis and nutrient dependency of cancer

**DOI:** 10.1186/s40880-018-0288-x

**Published:** 2018-04-25

**Authors:** Pranavi Koppula, Yilei Zhang, Li Zhuang, Boyi Gan

**Affiliations:** 1 Department of Experimental Radiation Oncology, The University of Texas MD Anderson Cancer Center, 1515 Holcombe Blvd, Houston, TX 77030 USA; 2 The University of Texas Graduate School of Biomedical Sciences, 6767 Bertner Ave., Houston, TX 77030 USA; 3 Department of Molecular and Cellular Oncology, The University of Texas MD Anderson Cancer Center, 1515 Holcombe Blvd, Houston, TX 77030 USA

**Keywords:** SLC7A11, xCT, System x_c_^−^, Cystine, Glutamate, Ferroptosis, Oxidative stress, Nutrient dependency, Cancer metabolism

## Abstract

Cancer cells often upregulate nutrient transporters to fulfill their increased biosynthetic and bioenergetic needs, and to maintain redox homeostasis. One nutrient transporter frequently overexpressed in human cancers is the cystine/glutamate antiporter solute carrier family 7 member 11 (SLC7A11; also known as xCT). SLC7A11 promotes cystine uptake and glutathione biosynthesis, resulting in protection from oxidative stress and ferroptotic cell death. Recent studies have unexpectedly revealed that SLC7A11 also plays critical roles in glutamine metabolism and regulates the glucose and glutamine dependency of cancer cells. This review discusses the roles of SLC7A11 in regulating the antioxidant response and nutrient dependency of cancer cells, explores our current understanding of SLC7A11 regulation in cancer metabolism, and highlights key open questions for future studies in this emerging research area. A deeper understanding of SLC7A11 in cancer metabolism may identify new therapeutic opportunities to target this important amino acid transporter for cancer treatment.

## Background

Metabolic flexibility was originally used to describe the ability of helminths to generate energy and critical metabolites via aerobic or anaerobic pathways as an adaptation to changes in environmental conditions [1]. Broadly, it refers to the capability of a biological system (organisms or cells) to adapt to metabolic changes in response to varying metabolic, environmental, or physical stimuli. At the cellular level, metabolic flexibility encompasses extensive rerouting of catabolic and anabolic pathways to maintain cellular homeostasis. For example, glucose and glutamine are principle nutrients that support biosynthetic and bioenergetic processes in most cells. Glucose limitation or impairment of mitochondrial pyruvate transport upregulates glutamine metabolism, which provides important metabolites to maintain the tricarboxylic acid (TCA) cycle and supports cell survival under glucose-limited conditions [2–4].

Cancer cells extensively reprogram their metabolic pathways to support increased biosynthetic and bioenergetic demands. One common mechanism employed by cancer cells for metabolic reprogramming is to increase uptake of nutrients critical for biosynthetic and bioenergetic processes in cancer cells, including glucose and amino acids such as glutamine [5, 6]. Cancer cells achieve this by mainly upregulating various transporters that mediate uptake of glucose and amino acids. Correspondingly, cancer cells may require certain nutrients for survival and thus have limited nutrient flexibility; i.e., some cancer cells undergo cell death when certain nutrients are limited, while under the same conditions, normal cells survive because they have more metabolic flexibility, which is commonly referred to as nutrient dependency of cancer cells. The mechanistic understanding of nutrient dependency in cancer cells may have important therapeutic implications for cancer treatment, because it suggests that drugs that impair nutrient metabolism may be effective for killing tumor cells dependent on corresponding nutrients for survival while sparing normal cells. One notable example of targeting nutrient dependency for cancer therapy is targeting asparagine in acute lymphoblastic leukemia (ALL). Whereas normal cells synthesize asparagine, ALL cells cannot synthesize asparagine because of their lack of asparagine synthase expression and thus are highly dependent on exogenous asparagine for survival. Based on this observation, asparaginase, the enzyme that converts asparagine to aspartic acid and ammonia, has been used in the clinic to treat ALL for decades [7].

Metabolic reprogramming in cancer cells also increases oxidative stress. To maintain the redox balance, cancer cells upregulate their antioxidant capabilities through a diverse array of mechanisms such as increasing biosynthesis of antioxidants including glutathione [8]. The generation of these antioxidants requires substantial supplies of carbon and cofactors (e.g. NADPH) from glucose and amino acids, which may limit nutrient flexibility and affect nutrient dependency of cancer cells. However, the mechanisms through which the antioxidant response regulates nutrient dependency in cancer cells remain largely unexplored. Solute carrier family 7 member 11 (SLC7A11; also known as xCT) is the light chain subunit of cystine/glutamate antiporter system x_c_^−^ and plays a vital role in maintaining redox homeostasis. Notably, recent studies have highlighted the emerging roles of SLC7A11 in regulating nutrient dependency of cancer cells. This review discusses the recent literature to understand the roles of SLC7A11 at the crossroads of reactive oxygen species (ROS) mitigation and nutrient dependency of cancer cells. We focus on SLC7A11 functions in the context of cancer biology in this review and refer to additional reviews discussing SLC7A11 functions in other pathologies and diseases [9–11].

## Structure and basic function of system x_c_^−^

**Fig. 1:**
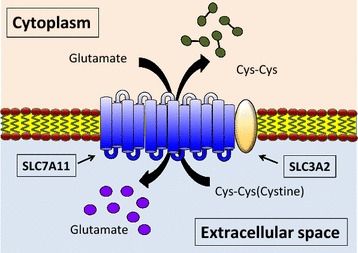
Structure and transport function of system x_c_^−^. System x_c_^−^ functions as a cystine/glutamate antiporter that imports one molecule of cystine in exchange for one molecule of intracellular glutamate. It is a heterodimer of the light chain subunit SLC7A11, which mediates transport activity of system x_c_^−^, and heavy chain subunit SLC3A2 that regulates trafficking of SLC7A11 to the plasma membrane

System x_c_^−^ is a sodium-independent antiporter of cystine and glutamate. This transporter system takes up extracellular cystine in exchange for intracellular glutamate at a 1:1 ratio [12] (Fig. 1). It consists of two subunits, the light chain subunit SLC7A11 and heavy chain subunit SLC3A2 (also known as CD98 or 4F2) (Fig. 1). In humans, the *SLC7A11* gene is located on chromosome 4 and includes 14 exons. *SLC7A11* is conserved across vertebrates, including zebrafish, but no obvious ortholog has been identified in other lower organisms such as *Caenorhabditis elegans*. SLC7A11 is a 12-pass transmembrane protein with both N- and C-termini located within the cytoplasm, whereas SLC3A2 is a single transmembrane protein with an intracellular N-terminus and heavily glycosylated extracellular domain as the C-terminus [13]. These two subunits are linked by a covalent disulfide bond. Efficient exchange of cystine and glutamate by system x_c_^−^ requires both heavy and light chain subunits. The light chain subunit SLC7A11 is responsible for the primary transport activity and is highly specific for cystine and glutamate, whereas the heavy chain subunit SLC3A2 primarily functions as a chaperone protein and is essential to regulate trafficking of SLC7A11 to the plasma membrane [14]. In addition, it has been shown that SLC3A2 deficiency results in a substantial decrease of SLC7A11 protein levels [15], suggesting that SLC3A2 is also required to maintain SLC7A11 protein stability.

**Fig. 2:**
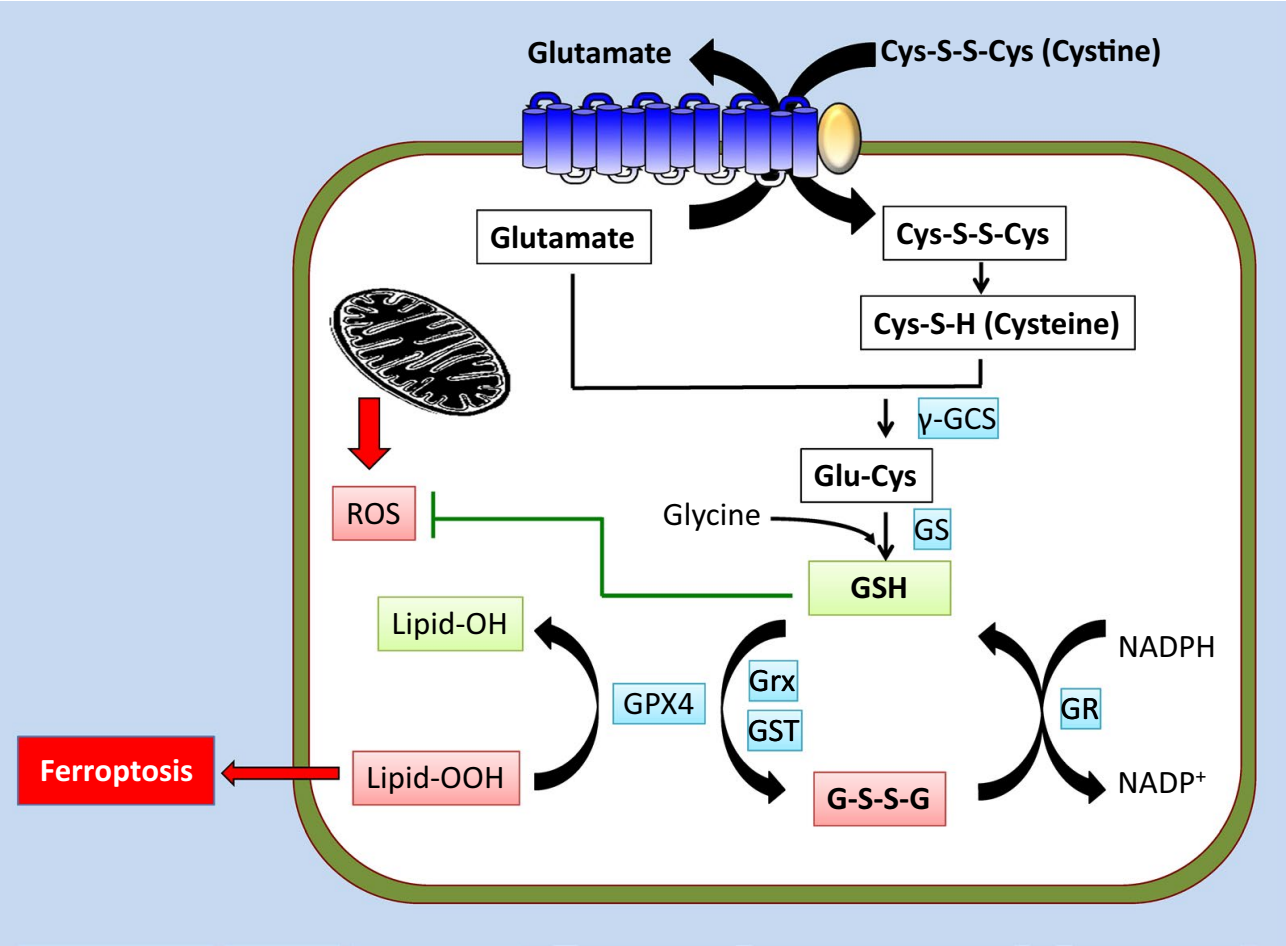
SLC7A11 promotes the oxidative stress response and inhibits ferroptosis. Extracellular cystine is imported into cells through SLC7A11 and converted to cysteine that in turn serves as the rate-limiting precursor for glutathione biosynthesis. Reduced glutathione (GSH) is used as a co-factor by various enzymes involved in ROS detoxification, such as GPX4. Overproduction of lipid hydroperoxides induces ferroptosis. GPX4 uses GSH to detoxify lipid hydroperoxides to lipid alcohols, thus repressing ferroptosis. *γGCS* γ-glutamylcysteine synthetase, *GS* glutathione synthetase, *GPX4* glutathione peroxidase 4, *GST* glutathione S-transferase, *GR* glutathione reductase, *Grx* glutaredoxin, *GSH* reduced glutathione, *GSSG* oxidized glutathione, *lipid-OOH* lipid hydroperoxide, *lipid-OH* lipid alcohol *Gly* glycine

System x_c_^−^ plays a central role in providing cysteine for biosynthesis of glutathione, a major antioxidant in cells [10] (Fig. 2). Although cysteine is synthesized from homocysteine and serine by the trans-sulfuration pathway in some tissues (e.g. the liver, kidney, and pancreas) and certain cell lines [16], most cells rely on system x_c_^−^ to take up cysteine from the extracellular environment. Because of the oxidizing conditions in the extracellular environment, extracellular cysteine is unstable and quickly oxidizes to cystine, a dimeric amino acid consisting of two cysteine molecules linked by a disulfide bond. Extracellular cystine is mainly transported into cells by system x_c_^−^ and then is quickly converted to cysteine because of the highly reducing conditions within cells. It should be noted that, if the extracellular space contains high levels of cysteine, cysteine can also be directly imported into cells by transporters such as system alanine–serine–cysteine (ASC). For example, bone marrow stromal cells surrounding chronic lymphocytic leukemia (CLL) cells secrete large amounts of cysteine, which are directly imported by ASC on the plasma membrane of CLL cells despite low expression of SLC7A11 in CLL cells [17].

Intracellular cysteine serves as a critical precursor for glutathione. Glutathione is a tripeptide of cysteine, glutamate, and glycine, among which cysteine is the rate-limiting precursor [18]. Glutathione biosynthesis involves a two step reaction [18] (Fig. 2). The first step is the rate-limiting reaction that generates γ-glutamyl-cysteine (γ-Glu-Cys) from cysteine and glutamate by the enzyme glutamate cysteine ligase (GCL; also called γ-glutamylcysteine synthetase). Then, in the second reaction, glycine is added to the C-terminus of γ-Glu-Cys to generate reduced glutathione (GSH) by the enzyme glutathione synthetase. Once synthesized, glutathione in its reduced form serves as a cofactor for ROS-detoxifying enzymes, such as glutathione peroxidase (GPX), which function to reduce peroxide-related products, such as hydrogen peroxide, at the expense of GSH and thus protects cells from ROS-induced damage. Through the GPX-mediated reaction, GSH is oxidized to its oxidized form (GSSG) that is recycled back to GSH by glutathione reductase (GR) at the expense of NADPH (Fig. 2).

In summary, system x_c_^−^ is a cystine/glutamate antiporter consisting of the transporter subunit SLC7A11 and regulatory subunit SLC3A2. System x_c_^−^-mediated transport of extracellular cystine is vital for appropriate maintenance of intracellular cysteine and GSH. Below, we discuss several other important functions that extend from this basic function of system x_c_^−^, including regulation of ferroptosis, the oxidative stress response, and nutrient dependency. It should be noted that SLC3A2 also serves as a chaperone protein for several other amino acid transporters such as large neutral amino acid transporter (LAT) 1, LAT2, and glucose transporter 1 [19–21]. Thus, SLC3A2 has pleiotropic functions beyond its function in system x_c_^−^. For this reason, most current studies of system x_c_^−^ have focused on SLC7A11. Therefore, we primarily discuss SLC7A11 in the following sections.

## SLC7A11 function in regulating ferroptosis and the oxidative stress response

Most regular culture conditions require cystine supplementation in the culture medium. It was observed many years ago that cystine deprivation in cell culture medium results in massive cell death of various cell lines, which is likely due to the depletion of intracellular GSH [10, 22]. However, the exact nature of cystine deprivation-induced cell death has remained elusive. In recent years, a new form of cell death termed ferroptosis was found to be associated with cystine depletion and impairment of system x_c_^−^-mediated cystine uptake [23, 24]. Specifically, ferroptosis is non-apoptotic cell death resulting from over-accumulation of lipid hydroperoxides in an iron-dependent manner. Accumulated lipid hydroperoxides in cells are normally detoxified by a GPX family member called glutathione peroxidase 4 (GPX4) that uses GSH to convert lipid hydroperoxides to lipid alcohols and thus represses ferroptosis [25] (Fig. 2). However, cystine deprivation or pharmacological inhibition of SLC7A11-mediated cystine uptake by drugs such as erastin results in depletion of intracellular GSH and induces ferroptotic cell death [23, 24]. Multiple lines of evidence support that ferroptosis is distinctive from other forms of cell death such as apoptosis [23, 24]. For example, morphologically, ferroptotic cells exhibit damaged shrunken mitochondria with an increased density but do not show obvious plasma membrane blebbing/rupture or DNA fragmentation in the nucleus, which are characteristics of apoptosis and necrosis. Biochemically, ferroptosis does not induce cleavage of caspase-3 or phosphorylation of receptor-interacting serine/threonine-protein kinase, the biochemical characteristics for apoptosis and necroptosis. Genetically, blockage of system x_c_^−^ transport activity by erastin treatment induces ferroptosis in *BAX*/*BAK*-deficient cells that do not undergo apoptosis. Ferroptosis can be prevented by ferroptosis inhibitor ferrostatin, but not by apoptosis or necroptosis inhibitors [23, 24]. Thus, by importing cystine and promoting GSH biosynthesis, SLC7A11 prevents accumulation of lipid hydroperoxides and protects cells from undergoing ferroptosis.

In addition to its role in ferroptosis inhibition, many studies have documented that SLC7A11 generally protects cells from cell death induced by various cellular stresses. For example, upregulation of *SLC7A11* in neuronal and cancer cell lines confers resistance to oxidative stress [26–28]. In addition, *SLC7A11* overexpression renders cancer cells more resistant to chemotherapy by temozolomide or cisplatin treatments [29, 30]. Conversely, it has been shown that inactivation of SLC7A11 by either small interfering RNA or pharmacological inhibition by sulfasalazine sensitizes cancer cells to proteasome inhibition [31]. It should be noted that other forms of cell death, such as apoptosis and necrosis, might be induced by these stress conditions. In most of these studies, the protective roles of SLC7A11 under stress conditions have been attributed to its functions to import cystine and promote GSH biosynthesis. In summary, it is well established that SLC7A11 has a pro-survival role through which SLC7A11-mediated cystine uptake helps cells to re-establish redox homeostasis in response to cellular stresses.

## SLC7A11 function in regulating nutrient dependency

In light of the established pro-survival function of SLC7A11 under stress conditions, it is surprising that several recent studies have revealed pro-death functions of SLC7A11 under glucose starvation [15, 32, 33]. Notably, these studies were conducted in a wide variety of cancer cell lines across various cancer types, including breast, cervical, kidney, and brain cancers, as well as mesothelioma, suggesting a universal role of SLC7A11 in regulating glucose starvation-induced cell death. In one study, *SLC7A11* was identified as a glucose starvation-induced gene [32]. Considering the protective role of SLC7A11 in response to oxidative stress [10, 24] and the close link between oxidative stress and glucose starvation-induced cell death [34–36], it was initially hypothesized that glucose starvation-induced *SLC7A11* expression serves as an adaptive response to detoxify glucose starvation-induced ROS and protect cells from glucose starvation. Unexpectedly, various lines of experimental evidence, including cell line correlations, *SLC7A11* overexpression, and SLC7A11 inactivation by knockdown or pharmacological inhibition, all indicate that SLC7A11 promotes cancer cell death under glucose starvation [32]. Another study conducted a loss-of-function screening to identify genes whose inactivation confers resistance to glucose starvation. Strikingly, this screening identified both SLC7A11 and SLC3A2 as top hits. Further experiments validated that SLC7A11 inactivation inhibits cancer cell death under glucose starvation, whereas its overexpression promotes such cell death [15].

**Fig. 3:**
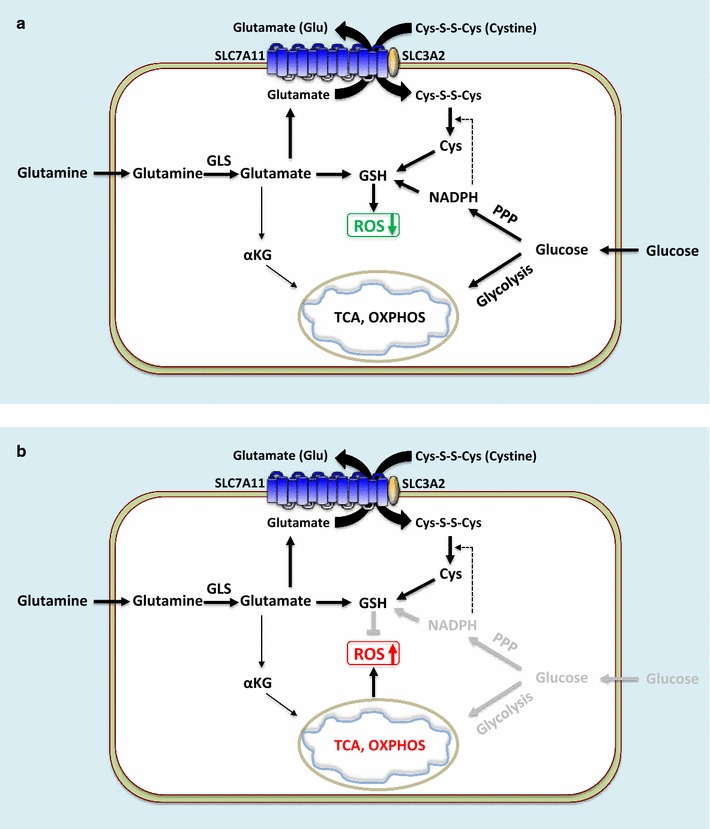
SLC7A11 regulates nutrient dependency of cancer cells. This schematic represents cells with high expression of *SLC7A11*. **a** Under normal conditions, SLC7A11 exports large amounts of intracellular glutamate in exchange for extracellular cystine. Cystine imported by SLC7A11 is converted to cysteine that supports glutathione biosynthesis and ROS detoxification. However, SLC7A11-mediated glutamate export limits intracellular glutamate supply to the TCA cycle and mitochondrial respiration, rendering such cells more dependent on glucose and/or glutamine supply for survival and growth. Glutamine is the major precursor for glutamate. Glucose provides the major carbon source for the TCA cycle as well as NADPH for glutathione biosynthesis and ROS detoxification. **b** Under glucose-deprived conditions, cells with high expression of *SLC7A11* lack adequate supplies to maintain the TCA cycle and mitochondrial respiration. In addition, cystine imported by SLC7A11 depletes NAPDH and induces ROS under glucose deprivation conditions, possibly because cystine conversion to cysteine consumes NADPH, which is largely provided by glucose. These events result in enhanced cell death of *SLC7A11*-high cancer cells under glucose starvation. *OXPHOS* oxidative phosphorylation, *PPP* pentose phosphate pathway, *GLS* glutaminase, *αKG* α-ketoglutarate, *Cys* cysteine

Both studies proposed that, because SLC7A11 exports large amounts of intracellular glutamate in exchange for extracellular cystine, cancer cells with high *SLC7A11* expression have more limited metabolic flexibility and are more dependent on glucose for survival. Accordingly, these cells are more sensitive to glucose starvation-induced cell death [15, 32] (Fig. 3). Several lines of evidence support this model. First, a previous study estimated that 30%–50% of intracellular glutamate is exported in exchange for extracellular cystine [37]. Consistent with this finding, *SLC7A11* knockdown leads to a significant increase of intracellular glutamate levels [15, 32]. In addition, glutamate is shunted into the TCA cycle through α-ketoglutarate (αKG) (Fig. 3). It has been shown that supplementation of αKG in cultures of cancer cells overexpressing *SLC7A11*, which have lower intracellular αKG levels, abrogates glucose starvation-induced cell death. Conversely, blockade of conversion from glutamate to αKG in cancer cells with *SLC7A11* knockdown, which have higher intracellular αKG levels, re-sensitizes these cells to glucose starvation [15, 32]. These data support a model in which *SLC7A11* deficiency promotes cell survival under glucose starvation by conserving intracellular glutamate to supply the TCA cycle (Fig. 3).

Recently, another study reported a similar observation that *SLC7A11* overexpression promotes cell death under glucose starvation [33]. However, this study proposed that cystine uptake, rather than glutamate export, underlies the increased sensitivity of cells with high expression of *SLC7A11* under glucose starvation (Fig. 3). It was shown that cystine is required for glucose starvation-induced cell death, and cystine uptake through SLC7A11 depletes intracellular NADPH and induces ROS under glucose starvation, thus sensitizing cells to glucose starvation-induced cell death [33]. This is rather surprising considering that cystine is also required for cell survival and often protects cells from oxidative stress by promoting glutathione synthesis (as discussed in the preceding section). How cystine plays opposing roles in regulating redox homeostasis under normal vs. glucose starvation conditions remains unknown. It is likely that both glutamate export and cystine uptake mediate SLC7A11 functions in regulating glucose dependency, or one mechanism over the other is employed in a context- and cell-line-dependent manner.

Glutamine is the most abundant amino acid in blood and cell culture medium. Once imported into cells, glutamine is converted to glutamate by glutaminase. Other studies have also identified a role of SLC7A11 in regulating glutamine dependency or sensitivity to glutaminase inhibition in cancer cells. A previous study has revealed that basal and claudin-low triple negative breast cancer (TNBC) cells tend to consume more glutamine and thus are more glutamine dependent compared with basal breast cancer cells and normal mammary epithelial cells [38]. Notably, the sensitivities of these cells to glutamine deprivation do not correlate with the expression levels of metabolic enzymes involved in glutamine metabolism, such as glutaminase and glutamine synthase, but correlate with the expression levels of SLC7A11 and cystine consumption. Specifically, basal and claudin-low TNBC cells generally exhibit higher SLC7A11 expression and more cysteine consumption than other cell lines. It was further shown that pharmacological inhibition of SLC7A11 by sulfasalazine attenuates the growth of xenografted tumors derived from these TNBC cells [38]. Such data indicate a model in which TNBCs with high *SLC7A11* expression are more dependent on glutamine for tumor growth, potentially because they need to consume more glutamine to maintain SLC7A11-mediated cystine/glutamate exchange, resulting in glutamine dependency (Fig. 3), and suggest SLC7A11 as a potential therapeutic target in TNBC.

Another recent study also uncovered a similar role of SLC7A11 in regulating glutamine dependency from a very different perspective by identifying environmental factors that cause differential dependencies on glutamine in cancer cells cultured in different media [39]. It was shown that cancer cells cultured in media that better represent in vivo conditions exhibit less glutamine metabolism and are less sensitive to glutaminase inhibition than the same cancer cells cultured in standard cell culture media. Further analyses identified cystine as the single nutrient accounting for the differential dependencies on glutamine in different culture conditions: high levels of cystine in standard cell culture media render cells more dependent on glutamine and more sensitive to glutaminase inhibition. It was further shown that environmental cystine regulates glutamine dependency through SLC7A11-mediated cystine/glutamate exchange [39]. Because glutamate is known to inhibit glutaminase activity [40], it was suggested that high levels of extracellular cystine deplete intracellular glutamate through SLC7A11-mediated cystine/glutamate exchange, resulting in glutaminase activation and glutamine dependency (Fig. 3).

Overall, a series of recent studies have identified an unexpected role of SLC7A11 in promoting cancer cell dependency on either glucose or glutamine. While one study proposes it is cystine uptake that mediates SLC7A11 functions in regulating glucose dependency by triggering cell death under glucose starvation [33], all other studies propose that glutamate export underlies SLC7A11-mediated increased sensitivity to glucose or glutamine starvation [15, 32, 38, 39]. Because cystine uptake and glutamate export are coupled by SLC7A11, it is difficult to clearly distinguish these two models, and it is likely that both mechanisms are employed in regulating nutrient dependency by SLC7A11.

## Molecular regulation of SLC7A11

**Fig. 4:**
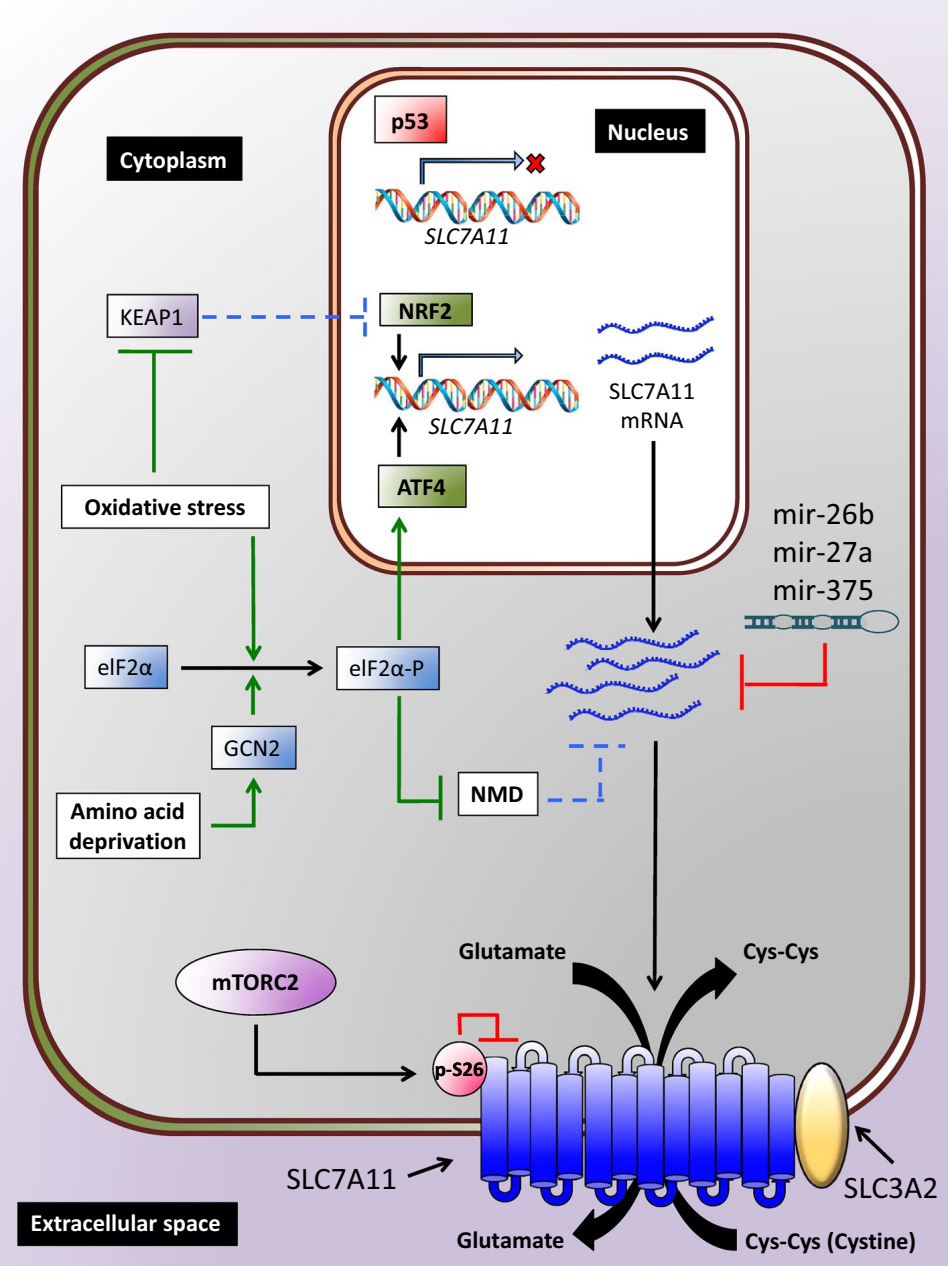
SLC7A11 regulation by transcriptional, post-transcriptional, and post-translational mechanisms. Cellular stresses, such as oxidative stress and amino acid starvation, induce *SLC7A11* transcription through NRF2 and/or ATF4 transcription factors, whereas p53 represses *SLC7A11* expression. *SLC7A11* mRNA stability can be negatively regulated by either microRNAs or NMD. Oxidative stress relieves NMD-mediated degradation of *SLC7A11* mRNA. mTORC2 phosphorylates SLC7A11 at serine 26, resulting in inhibition of SLC7A11 transport activity. *NMD* nonsense-mediated mRNA decay

Early studies documented that various stress conditions induce the cystine/glutamate antiporter activity of system x_c_^−^, such as amino acid deprivation (including cystine deprivation), electrophilic agents, and oxidative stress [41–43]. Later studies revealed that these stress conditions also induce *SLC7A11* expression [44, 45], which provide mechanistic insights into stress-induced system x_c_^−^ activity. In addition, a recent study showed that glucose starvation induces *SLC7A11* expression [32], although whether glucose starvation also induces the transport activity of system x_c_^−^ remains less clear [44]. It has been proposed that stress-induced *SLC7A11* expression and system x_c_^−^ activity generally serve as an adaptive response to import more cystine and re-establish the redox balance in response to stress stimuli. Extensive studies have identified two transcription factors that regulate stress-induced *SLC7A11* transcription, namely nuclear factor erythroid 2-related factor 2 (NRF2) and activating transcription factor 4 (ATF4) (Fig. 4), which are discussed below.

NRF2 is a master transcription factor that mediates the antioxidant response. Under basal unstressed conditions, NRF2 interacts with kelch-like ECH-associated protein-1 (KEAP1), a substrate adaptor protein for the Cullin3-dependent ubiquitin ligase complex, and is targeted for KEAP1-Cullin3-mediated polyubiquitination and proteasomal degradation [46]. Oxidative stress inducers, such as oxidants and electrophiles, induce oxidation of the reactive cysteine residues on KEAP1, resulting in the impairment of NRF2 degradation by the KEAP1-Cullin3 ubiquitin ligase complex. Subsequently, the stabilized NRF2 translocates into the nucleus, binds to antioxidant response elements (AREs) in gene promoter regions, and regulates the transcription of a host of target genes involved in the antioxidant response and cellular redox maintenance, including *GCL* and *GR* discussed in the previous section [46]. Analysis of the *SLC7A11* promoter identified several AREs. Further analysis revealed that the induction of *SLC7A11* expression by electrophilic agents and other cellular stresses is mediated by NRF2 binding to AREs in the *SLC7A11* promoter [45]. Correspondingly, it has been shown that overexpression of *NRF2* upregulates the expression of *SLC7A11*, among other antioxidant target genes, leading to increased GSH biosynthesis [47]. Thus, *SLC7A11* is one of the NRF2 transcriptional targets that mediate the antioxidant response (Fig. 4).

ATF4 is a transcription factor that regulates redox homeostasis, amino acid metabolism, and endoplasmic reticulum (ER) stress [48]. In contrast to NRF2, which stabilizes in response to stress, translation of *ATF4* mRNA is enhanced under various stress conditions. Under unstressed conditions, *ATF4* mRNA translation is repressed by the presence of upstream open reading frames (uORFs) located in the 5′-untranslated region of *ATF4* mRNA. Eukaryotic initiation factor 2α (eIF2α) is phosphorylated and inhibited by several upstream kinases that are activated by various types of cellular stresses, such as amino acid deprivation, ER stress, and viral infection [48]. Phosphorylated eIF2α then inhibits the translation of many mRNAs, including *ATF4* uORFs, thus liberating *ATF4* mRNA translation and resulting in an increase of ATF4 protein levels [48]. One upstream kinase of eIF2α is general control non-derepressible-2 (GCN2) that is activated by free tRNAs under amino acid deprivation. Thus, amino acid deprivation activates GCN2, which then phosphorylates and inactivates eIF2α, leading to increased ATF4 protein synthesis. Subsequently, ATF4 binds to amino acid response elements (AAREs) in gene promoter regions and regulates the transcription of many genes involved in amino acid metabolism and the stress response to adapt to amino acid starvation [49]. It has been shown that deprivation of different amino acids, most notably cystine, induces *SLC7A11* expression, and amino acid starvation-induced *SLC7A11* expression requires ATF4 binding to AAREs in the *SLC7A11* promoter [44]. In addition, expression of a non-phosphorylatable eIF2α mutant, which cannot be phosphorylated by GCN2, in mouse embryonic fibroblasts results in decreases of ATF4 expression, *SLC7A11* promoter activity, and system x_c_^−^ activity under stress conditions [50]. Taken together, these data support a model in which amino acid deprivation induces *SLC7A11* expression through a GCN2-eIF2α-ATF4 signaling axis (Fig. 4).

Recent studies suggest a model in which NRF2 and ATF4 cooperatively regulate *SLC7A11* expression under stress conditions. It has been shown that NRF2 and ATF4 interact with each other and coordinately regulate *SLC7A11* expression [31]. Correspondingly, the induction of *SLC7A11* expression by various stress conditions, such as proteasome inhibitor treatment or glucose starvation, requires both NRF2 and ATF4 [31, 32]. Furthermore, the tumor suppressor p53 was identified as another transcription factor that regulates *SLC7A11* expression [51]. In contrast to ATF4 and NRF2 that upregulate *SLC7A11* transcription, p53 represses *SLC7A11* expression [51] (Fig. 4). However, it is unclear whether p53 regulates *SLC7A11* expression under any stress condition.

Multiple studies have revealed that, through regulating *SLC7A11* expression, these aforementioned transcription factors modulate downstream biological effects mediated by SLC7A11, including ferroptotic cell death, stress resistance, and nutrient dependency. As discussed above, SLC7A11 inhibits ferroptosis by importing cystine and promoting GSH biosynthesis [24]. Correspondingly, it has been shown that ATF4 and NRF2 inhibit ferroptosis at least partly through upregulating *SLC7A11* expression, whereas p53 promotes ferroptosis by repressing *SLC7A11* expression [51–55]. In addition, it has been shown that ATF4 and NRF2 promote resistance to various cellular stresses, including oxidative stress, genotoxic stress induced by chemotherapy, and proteasome inhibition, at least partly through SLC7A11 [26, 31, 56, 57], which is in line with the similar protective functions of SLC7A11 in mediating these stress responses as discussed in the previous section. Recent studies have indicated that NRF2 and ATF4 regulate cancer cell dependency on either glucose or glutamine through SLC7A11. Specifically, it has been shown that deficiency of *ATF4* or *NRF2* expression decreases SLC7A11 expression, and similar to SLC7A11 deficiency, improves cancer cell survival under glucose starvation. Importantly, restoration of *SLC7A11* expression in ATF4- or NRF2-deficient cells re-sensitizes cells to glucose starvation [15, 32]. Another recent study showed that NRF2 activation in cancer cells by either *KEAP1* mutation or pharmacological stimulation leads to decreased intracellular glutamate pools at least partly through SLC7A11-mediated glutamate export, resulting in enhanced sensitivity to glutamine starvation and glutaminase inhibition [58].

While most current studies have focused on transcriptional regulation of SLC7A11 by stress signaling, stress-induced *SLC7A11* mRNA levels are also regulated by post-transcriptional mechanisms. Nonsense-mediated mRNA decay (NMD) is a surveillance pathway that degrades mRNAs with premature stop codons as well as non-mutated mRNAs that often encode proteins involved in stress responses [59]. *SLC7A11* mRNA is subjected to degradation by NMD, and various cellular stresses, including amino acid deprivation, inhibit NMD-induced *SLC7A11* mRNA degradation, resulting in *SLC7A11* mRNA stabilization, increased SLC7A11 protein levels, and enhanced cysteine transport and GSH synthesis [60] (Fig. 4). Other post-transcriptional mechanisms governing *SLC7A11* mRNA levels include SLC7A11 regulation by microRNAs including miR-27a, miR-26b, and miR-375 [61–63] (Fig. 4). These microRNAs directly target SLC7A11 and suppress *SLC7A11* mRNA stability and/or translation. Overexpression of these microRNAs compromises cell viability, proliferation, and invasion of various cancer cells, likely through *SLC7A11* repression, and they are downregulated in various human cancers, suggesting tumor suppressive functions of these microRNAs [61–63].

Emerging evidence also indicates that SLC7A11 is regulated by interactions with other proteins or post-translational modifications. As discussed above, SLC3A2, the obligate partner of SLC7A11, is required to maintain SLC7A11 protein stability [15]. Another adhesion molecule, CD44 variant (CD44v), was also identified as a SLC7A11-interacting protein that maintains SLC7A11 protein stability [64]. CD44v deficiency compromises both the stability and cell surface localization of SLC7A11, resulting in depletion of intracellular GSH, ROS induction, and attenuation of gastric tumor development [64]. A very recent study identified phosphorylation as a regulatory mechanism of SLC7A11 functions [65]. Mammalian target of rapamycin complex 2 (mTORC2; also known as mechanistic target of rapamycin complex 2) is a serine/threonine kinase consisting of multiple protein components, including mTOR, and functions to integrate upstream growth factor stimulation with cellular processes such as cell survival by phosphorylating various downstream targets [66]. mTORC2 components were identified as binding proteins of SLC7A11 through mass spectrometric analysis. Further analyses revealed that, in response to growth factor stimulation, mTORC2 phosphorylates serine 26 located at the N-terminal cytoplasmic tail of SLC7A11, and this phosphorylation inhibits the transport activity of SLC7A11 [65] (Fig. 4).

Taken together, cellular stresses that impair redox homeostasis often induce the transport activity of system x_c_^−^ at least partly by upregulating *SLC7A11* mRNA levels, which can be controlled by either inducing ATF4/NRF2-mediated *SLC7A11* transcription or inhibiting NMD-mediated *SLC7A11* mRNA degradation under stress conditions (Fig. 4). Whether stress can modulate SLC7A11 through any post-translational mechanism remains largely unknown.

## Conclusion and future perspectives

Cancer cells often encounter increased oxidative stress due to their altered metabolic programs as well as changing microenvironments that induce ROS. Consequently, cancer cells upregulate their antioxidant capabilities to maintain redox homeostasis [8]. One strategy employed by cancer cells is to upregulate *SLC7A11* expression and thus SLC7A11-mediated cystine uptake, which allows cancer cells to have better capabilities to detoxify ROS as well as grow and survive under oxidative stress conditions. However, emerging evidence suggests that high expression of *SLC7A11* in cancer cells also renders them highly dependent on glucose and/or glutamine [15, 32, 33, 39, 58]. Thus, it appears that SLC7A11 acts a double-edged sword in regulating the redox balance and nutrient dependency of cancer cells. Currently, we are only beginning to understand the roles of this amino acid transporter in cancer metabolism. Here, we outline and discuss several important questions that merit further investigation in future studies. (i) How does SLC7A11 regulate nutrient dependency in cancer cells? (ii) What are other regulatory mechanisms to control SLC7A11 functions under metabolic stress conditions? (iii) What is the exact role of SLC7A11 in cancer development? (iv) How can SLC7A11 be targeted for cancer prevention or treatment?

Several open questions remain concerning the roles of SLC7A11 in regulating nutrient dependency. First, while recent studies suggest that high expression of *SLC7A11* renders cancer cells more dependent on glutamine for growth [38, 39, 58], another study has documented that *SLC7A11* overexpression or knockdown do not affect cancer cell viability under glutamine deprivation, suggesting that SLC7A11 does not regulate cancer cell dependency on glutamine for survival [15]. Because these studies were conducted in different cancer cell lines and employed different assays (cell growth vs. cell viability assays), it is possible that SLC7A11 regulates glutamine dependency in a cell-line-dependent manner, or alternatively, SLC7A11 promotes cancer cell dependency on glutamine for growth but not survival. In contrast, SLC7A11 promotes cancer cell dependency on glucose mainly for survival. In addition, because deprivation of glucose or glutamine induces oxidative stress [32, 67], how SLC7A11 balances its opposing roles in protecting against oxidative stress vs. inhibiting cell survival/growth under glucose or glutamine deprivation remains less clear. Because cystine deprivation often induces ferroptotic cell death [23], it is counterintuitive that cystine depletion also protects cells from glucose starvation-induced cell death [33]. How cystine exerts opposite effects on cell death/survival under normal and glucose starvation conditions remains unclear. Further studies will be needed to address these interesting issues.

To maintain redox homeostasis, mammalian cells have evolved multiple elegant systems, including transcriptional, post-transcriptional, and post-translational mechanisms, to fine-tune cellular responses to oxidative stress. Most current studies have focused on transcriptional regulation of SLC7A11 under stress conditions. It will be interesting to continue to examine whether other transcription factors involved in cancer metabolism, such as Myc, also regulate SLC7A11 and SLC7A11-mediated redox homeostasis. In addition, whether SLC7A11 is modulated through other mechanisms, particularly post-translational modifications, remains largely unknown. Thus far, the recent study on SLC7A11 phosphorylation by mTORC2 is the only example indicating that SLC7A11-mediated transport activity is modulated by a post-translational mechanism [65]. Further studies will be aimed at understanding whether SLC7A11 protein stability, subcellular localization, and transport activity can be modulated by any post-translational modification or its interactions with other proteins, and whether such regulatory mechanisms in turn affect downstream biological effects regulated by SLC7A11.

The established roles of SLC7A11 in ferroptosis inhibition and protecting cells from oxidative stress suggest a tumor-promoting function of SLC7A11. Consistent with this notion, pharmacological inhibition or knockdown of *SLC7A11* inhibits xenografted tumor development ([38] and our unpublished observation). However, the recent findings of SLC7A11 functions in nutrient dependency [15, 32, 33, 39, 58] suggest complex roles of SLC7A11 in cancer development. Specifically, glucose and glutamine supplies are often limited in tumor cells within a tumor mass because of poor tumor vasculature in the tumor microenvironment [68]. Thus, tumor cells with high expression of SLC7A11 within established tumors may have limited capabilities to grow and survive, because they would be presumably more sensitive to glucose- and/or glutamine-limited conditions. Based on this scenario, it is possible that *SLC7A11* overexpression promotes tumor initiation, but once the tumor is established, *SLC7A11*-high tumor cells might be vulnerable to metabolic stress induced by glucose or glutamine deprivation in the tumor microenvironment, resulting in limited tumor progression. This hypothesis predicts an opposing role of SLC7A11 in tumor initiation and maintenance. Genetically engineered mouse models (GEMMs) represent the best model systems to further test this hypothesis. An *Slc7a11* knockout (KO) mouse model is already available [69]. However, there have been essentially no publications using GEMMs to study SLC7A11 functions in cancer. We envision that many future studies will use GEMMs (*Slc7a11* KO and transgenic mouse models) to investigate SLC7A11 in diverse cancers, which will undoubtedly provide key insights into SLC7A11 functions in cancer development.

*SLC7A11* exhibits restricted expression patterns in normal tissues with primary expression in the brain [9, 70–72]. It is highly expressed in various human cancers [11, 73], although the mutation frequency of the *SLC7A11* gene in human cancers is generally low (analysis from the cBioPortal for Cancer Genomics). In addition, *Slc7a11* KO mice are viable and fertile with no obvious phenotype [69]. Considering the critical role of GSH biosynthesis in physiology, it remains less understood why SLC7A11 is dispensable for normal development in vivo. It has been suggested that SLC7A11 functions are compensated by a different cystine transport system in vivo [69]. However, the restricted expression patterns of *SLC7A11* in normal tissues and the dispensability of SLC7A11 in normal development suggest that SLC7A11 might be an ideal therapeutic target for treating cancers with high SLC7A11 expression. Various compounds and drugs have been identified to block the transport activity of SLC7A11 [9], among which the most prominent example is sulfasalazine, an FDA-approved drug commonly used to treat chronic inflammatory diseases such as rheumatoid arthritis [74]. Another commonly used compound to block SLC7A11-mediated cystine transport and induce ferroptosis is erastin [24]. Correspondingly, it has been shown that treatment with sulfasalazine or erastin reduces tumor development [25, 38], suggesting therapeutic effects of these drugs for cancer treatment. However, all current available SLC7A11 inhibitors, including sulfasalazine and erastin, have off-target effects, thereby limiting their use as SLC7A11-specific inhibitors in clinical settings. The development of highly specific SLC7A11 inhibitors will be critical to target SLC7A11 for cancer treatment.

Notably, recent findings regarding SLC7A11 functions in regulating nutrient dependency suggest another strategy to target tumors with high *SLC7A11* expression. Specifically, it has been proposed that, because cancer cells with high *SLC7A11* expression are dependent on glucose or glutamine for survival and growth, such tumors may be sensitive to drugs that block glucose or glutamine metabolism [32, 58]. In support of this hypothesis, a recent study has shown that lung cancer cells and lung tumors with *KEAP1* mutations exhibit constitutive NRF2 activation and are sensitive to glutaminase inhibition, which is likely due to the high expression of *SLC7A11* in these tumors, suggesting the use of glutaminase inhibitors to treat lung cancer patients with *KEAP1* mutations [75]. Future studies on SLC7A11 functions in regulating nutrient dependency may provide novel and effective therapeutic strategies to treat a subset of cancer patients with *SLC7A11* overexpression.
